# Occurrence and characteristics of extended-spectrum β-lactamase (ESBL) producing *Enterobacteriaceae *in food producing animals, minced meat and raw milk

**DOI:** 10.1186/1746-6148-8-21

**Published:** 2012-03-07

**Authors:** Nadine Geser, Roger Stephan, Herbert Hächler

**Affiliations:** 1 Institute for Food Safety and Hygiene, Vetsuisse Faculty, University of Zurich, CH-8057 Zurich, Switzerland

## Abstract

**Background:**

The impact of food animals as a possible reservoir for extended-spectrum beta-lactamase (ESBL) producing *Enterobacteriaceae*, and the dissemination of such strains into the food production chain need to be assessed. In this study 334 fecal samples from pigs, cattle, chicken and sheep were investigated at slaughter. Additionally, 100 raw milk samples, representing bulk tank milk of 100 different dairy farms, 104 minced meat (pork and beef) samples and 67 *E. coli *isolates from cattle *E. coli *mastitis were analyzed.

**Results:**

As many as 15.3% of the porcine, 13.7% of the bovine, 8.6% of the sheep and 63.4% of the chicken fecal samples yielded ESBL producers after an enrichment step. In contrast, none of the minced meat, none of the bulk tank milk samples and only one of the mastitis milk samples contained ESBL producing strains. Of the total of 91 isolates, 89 were *E. coli*, one was *Citrobacter youngae *and one was *Enterobacter cloacae*. PCR analysis revealed that 78 isolates (85.7%) produced CTX-M group 1 ESBLs while six isolates (6.6%) produced CTX-M group 9 enzymes. Five detected ESBLs (5.5%) belonged to the SHV group and 2 isolates (2.2%) contained a TEM-type enzyme. A total of 27 CTX-M producers were additionally PCR-positive for TEM-beta-lactamase. The ESBL-encoding genes of 53 isolates were sequenced of which 34 produced CTX-M-1, 6 produced CTX-M-14, 5 produced CTX-M-15 and also 5 produced SHV-12. Two isolates produced TEM-52 and one isolate expressed a novel CTX-M group 1 ESBL, CTX-M-117. One isolate--aside from a CTX-M ESBL-- contained an additional novel TEM-type broad-spectrum beta-lactamase, TEM-186.

**Conclusions:**

The relatively high rates of ESBL producers in food animals and the high genetic diversity among these isolates are worrisome and indicate an established reservoir in farm animals.

## Background

Antimicrobial resistance in bacteria has emerged as a problem in both human and veterinary medicine. One of the currently most important resistance mechanisms in *Enterobacteriaceae*, which reduces the efficacy even of modern expanded-spectrum cephalosporins (except cephamycins and carbapenems) and monobactams is based on plasmid-mediated production of enzymes that inactivate these compounds by hydrolyzing their β-lactam ring. Such resistance is encoded by an increasing number of different point-mutational variants of classical broad-spectrum β-lactamases (BSBL). These variants are called extended spectrum β-lactamases (ESBL): most are derivates of TEM and SHV β-lactamase families, whereas other groups, such as CTX-M, PER and KPC β-lactamases have been described more recently [1]. The phenotypical difference between BSBLs and ESBLs is that the latter efficiently hydrolyze 3rd- and 4th-generation cephalosporins, additionally to penicillins and lower generation cephalosporins as the BSBLs are capable of. BSBLs and ESBLs are inhibited by clavulanic acid, sulbactam and tazobactam [2], a feature that is used (i) as a criterion for classification of β-lactamases and (ii) for diagnostic ESBL detection purposes. Until now more than 600 ESBL variants are known http://www.lahey.org/Studies/ (last accessed January 2012). Among them, the over 100 CTX-M enzymes so far reported may be grouped into five main subgroups. Each of them is characterized by a group-representative single structure according to their amino acid sequence (group 1: CTX-M-1, group 2: CTX-M-2, group 8: CTX-M-8, group 9: CTX-M-9, and group 25: CTX-M-25) [3]. As a matter of growing concern, resistance caused by ESBLs is often associated with resistance to other classes of antibiotics like fluoroquinolones, aminoglycosides and trimethoprim-sulfmethoxazole [1,4].

Since the first description of ESBL producing *Enterobacteriaceae *isolated from hospitalized humans [5], many nosocomial outbreaks have been reported. However, since a few years, there is an increase in the detection of ESBL producing strains in the community [6]. More recently, reports have also raised concern about the dissemination of ESBL producing *E. coli *in healthy food producing animals in several countries in Europe [7-9] and Asia [10,11] or in food products like meat, fish and raw milk [12-14]. Recently, Wittum et al. [15] and Doi et al. [16] described for the first time ESBL producers in healthy dairy cattle and retail meat in the USA.

Therefore, the impact of healthy farm animals as a possible reservoir for ESBL producing *Enterobacteriaceae *on the food processing chain has to be assessed. The aim of the present study was to screen for the occurrence of ESBL producing *Enterobacteriaceae *in healthy swine, cattle, sheep and chicken at slaughter as well as in milk and meat in Switzerland and to further characterize isolates.

## Results

After an enrichment step ESBL producers were isolated from 90 (26.9%) of the investigated 334 fecal samples, and one ESBL producer (1.5%) was found in 67 *E. coli *mastitis milk isolates, but none was isolated from either minced meat (pork and beef) or bulk tank milk samples. The ESBL prevalence among cattle was 13.7%, 25.3% among calves (animals under 6 months), 8.6% among sheep, and 15.3% among pigs. For chickens (herd level) a very high prevalence of 63.4% was determined (Table 1). All suspected isolates were phenotypically confirmed, in that they showed a synergy effect with at least 1 of 3 strips when tested with Etest-ESBL strips containing cefepime, cefotaxime or ceftazidime, and they yielded factors > 8 when ratios of MIC (cephalosporin)/MIC (cephalosporin plus clavulanic acid) were calculated.

**Table 1:** Occurrence of ESBL producers in food-producing animals at slaughter as well as in minced meat, bulk tank milk and isolates from bovine mastitis in Switzerland

Origin		n	Number of samples with ESBL producers (percentage)
Cattle, fecal samples		124	17 (13.7%; [95% CI, 8.1; 21.0])

	calves	63	16 (25.3%; [95% CI, 15.3; 37.9])

	others	61	1 (1.6%; [95% CI, 0.4; 8.7])

Pig, fecal samples		59	9 (15.3%; [95% CI, 7.2; 26.9])

Chicken, fecal samples from crates of different flocks		93	59 (63.4%; [95% CI, 52.8; 73.2])

Sheep, fecal samples		58	5 (8.6%; [95% CI, 2.9; 18.9])

	lambs	40	2 (5.0%; [95% CI, 0.6; 16.9])

	others	18	3 (16.7%; [95% CI, 3.5; 41.4])

Mined meat (pork, beef)		104	0 (0.0%; [95% CI, 0.0; 3.4])

Bulk tank milk		100	0 (0.0%; [95% CI, 0.0; 3.6])

*E. coli *isolates from mastitis milk		67	1 (1.5%; [95% CI, 0.3; 8.0])

n: number of samples tested

CI: confidence interval

Almost all isolated ESBL producers were *E. coli *(89 out of 91), the exceptions being one *Enterobacter cloacae *isolated from a sheep, and one *Citrobacter youngae *isolated from a calf (Table 2).

**Table 2:** Identification and further characterization of the 91 ESBL producers isolated from 334 healthy food-producing animals at slaughter and from 67 *Escherichia coli *mastitis milk samples in Switzerland

Sample number	Origin	Species	Expressed ESBL & accompanying β- lactamase	β-lactam antibiotic resistances										Additional resistance
				**AM**	**AMC**	**CF**	**CXM**	**CPD**	**CTX**	**CAZ**	**FEP**	**FOX**	**IPM**	

13	pig	*E. coli*	CTX-M-1	r	s	r	r	r	i^a^	s^a^	s^a^	ss	s	NA. S, SXT, TE

14	pig	*E. coli*	CTX-M-1	r	s	r	r	r	i^a^	s^a^	i^a^	s	s	NA, TE

64	pig	*E. coli*	CTX-M-1	r	s	r	r	r	r	s^a^	s^a^	s	s	SXT

65	pig	*E. coli*	CTX-M-1	r	s	r	r	r	i^a^	s^a^	s^a^	s	s	NA, TE

17	pig	*E. coli*	CTX-M-1 & TEM-1	r	s	r	r	r	r	s^a^	s^a^	s	s	CIP, NA, S, TE

18	pig	*E. coli*	CTX-M-1 & TEM-1	r	s	r	r	r	r	s^a^	i^a^	s	s	C, CIP, NA, S, SXT, TE

72	pig	*E. coli*	CTX-M-1 & TEM-1	r	s	r	r	r	i^a^	s^a^	s^a^	s	s	S, SXT

60	pig	*E. coli*	CTX-M-1 & TEM-186	r	s	r	r	r	i^a^	s^a^	s^a^	s	s	S, SXT

16	pig	*E. coli*	CTX-M-14 & TEM-1	r	s	r	r	r	r	s^a^	s^a^	s	s	CIP, GM, NA, S, SXT, TE

46	calf	*E. coli*	CTX-M-1	r	s	r	r	r	i^a^	s^a^	s^a^	s	s	C, GM, S, SXT, TE

112	calf	*E. coli*	CTX-M-1	r	s	r	r	r	r	s^a^	s^a^	s	s	C, GM, S, SXT, TE

114	calf	*E. coli*	CTX-M-1	r	s	r	r	r	r	s^a^	s^a^	s	s	C, GM, S, SXT, TE

142.09_b	calf	*E. coli*	CTX-M-1	r	s	r	r	r	r	s^a^	s^a^	s	s	C, GM, S, SXT, TE

142.09_g	calf	*C. youngae*	CTX-M-1	r	s	r	r	r	r	s^a^	s^a^	s	s	C, GM, S, SXT, TE

68	young cow	*E. coli*	CTX-M-1	r	s	r	r	r	r	s^a^	s^a^	s	s	NA

128	calf	*E. coli*	CTX-M-1	r	s	r	r	r	i^a^	s^a^	r	s	s	S, SXT, TE

136	calf	*E. coli*	CTX-M-1	r	s	r	r	r	r	s^a^	s^a^	s	s	CIP, NA, TE

129	calf	*E. coli*	CTX-M-1 & TEM-1	r	s	r	r	r	i^a^	s^a^	s^a^	s	s	S, SXT, TE

104	calf	*E. coli*	CTX-M-1 & TEM-1	r	s	r	r	r	i^a^	s^a^	s^a^	s	s	C, GM, S, SXT, TE

47	calf	*E. coli*	CTX-M-15	r	s	r	r	r	r	i^a^	s^a^	s	s	C, GM, S, SXT, TE

52	calf	*E. coli*	CTX-M-15	r	s	r	r	r	r	i^a^	r	s	s	CIP, NA, GM, SXT, TE

53	calf	*E. coli*	CTX-M-15	r	i	r	r	r	r	i^a^	i^a^	s	s	CIP, NA

124	calf	*E. coli*	CTX-M-15	r	s	r	r	r	r	r	s^a^	s	s	CIP, NA, S

142.11_n	calf	*E. coli*	CTX-M-117 & TEM-1	r	s	r	r	r	i^a^	s^a^	s^a^	s	s	C, CIP, GM, NA, S, SXT, TE

115	calf	*E. coli*	CTX-M-14 & TEM-1	r	i	r	r	r	i^a^	s^a^	s^a^	s	s	C, CIP, NA, SXT

116	calf	*E. coli*	CTX-M-14 & TEM-1	r	i	r	r	r	i^a^	s^a^	s^a^	s	s	C, CIP, GM, NA, S, SXT, TE

2	lamb	*E. coli*	CTX-M-1 & TEM-1	r	s	r	r	r	r	s^a^	s^a^	s	s	C, NA, S, TE

108	sheep	*E. coli*	CTX-M-15 & TEM-1	r	i	r	r	r	r	i^a^	i^a^	s	s	CIP, GM, S, SXT, TE

100	sheep	*E. coli*	CTX-M-14 & TEM-1	r	s	r	r	r	i^a^	s^a^	s^a^	s	s	C, CIP, GM, NA, S, TE

102	sheep	*E. coli*	CTX-M-14 & TEM-1	r	i	r	r	r	i^a^	s^a^	s^a^	s	s	C, CIP, GM, NA, S, TE

11	lamb	*E. cloacae*	SHV-12 & TEM-1	r	r	r	r	r	i^a^	r	s^a^	r	s	C, S, SXT, TE

3	chicken	*E. coli*	CTX-M group 1^+^	r	s	r	r	r	i^a^	s^a^	s^a^	s	s	NA, TE

4	chicken	*E. coli*	CTX-M group 1^+^	r	s	r	r	r	r	s^a^	s^a^	s	s	TE

8	chicken	*E. coli*	CTX-M group 1^+^	r	s	r	r	r	i^a^	s^a^	s^a^	s	s	SXT, TE

10	chicken	*E. coli*	CTX-M group 1^+^	r	s	r	r	r	r	s^a^	s^a^	s	s	S, SXT, TE

12	chicken	*E. coli*	CTX-M group 1^+^	r	s	r	r	r	r	s^a^	s^a^	s	s	NA, SXT, TE

16	chicken	*E. coli*	CTX-M group 1^+^	r	s	r	r	r	i^a^	s^a^	s^a^	s	s	NA, TE

17	chicken	*E. coli*	CTX-M-1	r	s	r	r	r	r	s^a^	s^a^	s	s	NA, TE

26	chicken	*E. coli*	CTX-M group 1^+^	r	s	r	r	r	i^a^	s^a^	s^a^	s	s	TE

30	chicken	*E. coli*	CTX-M group 1^+^	r	s	r	r	r	i^a^	s^a^	s^a^	s	s	TE

31	chicken	*E. coli*	CTX-M-1	r	s	r	r	r	i^a^	s^a^	s^a^	s	s	SXT, TE

33	chicken	*E. coli*	CTX-M group 1^+^	r	s	r	r	r	r	s^a^	i^a^	s	s	NA, TE

35	chicken	*E. coli*	CTX-M-1	r	s	r	r	r	i^a^	r	s^a^	s	s	NA, SXT, TE

40	chicken	*E. coli*	CTX-M group 1^+^	r	s	r	r	r	i^a^	s^a^	s^a^	s	s	SXT, TE

41	chicken	*E. coli*	CTX-M group 1^+^	r	s	r	r	r	r	s^a^	s^a^	s	s	SXT

42	chicken	*E. coli*	CTX-M group 1^+^	r	s	r	r	r	i^a^	s^a^	s^a^	s	s	NA, TE

43	chicken	*E. coli*	CTX-M group 1^+^	r	s	r	r	r	i^a^	s^a^	s^a^	s	s	SXT, TE

44	chicken	*E. coli*	CTX-M group 1^+^	r	s	r	r	r	i^a^	s^a^	s^a^	s	s	NA, SXT, TE

45	chicken	*E. coli*	CTX-M group 1^+^	r	s	r	r	r	i^a^	s^a^	s^a^	s	s	NA, SXT

46	chicken	*E. coli*	CTX-M group 1^+^	r	s	r	r	r	i^a^	s^a^	s^a^	s	s	SXT, TE

47	chicken	*E. coli*	CTX-M-1	r	s	r	r	r	r	s^a^	s^a^	s	s	S, SXT, TE

48	chicken	*E. coli*	CTX-M group 1^+^	r	s	r	r	r	r	s^a^	s^a^	s	s	SXT, TE

49	chicken	*E. coli*	CTX-M-1	r	s	r	r	r	i^a^	s^a^	s^a^	s	s	S, SXT, TE

51	chicken	*E. coli*	CTX-M group 1^+^	r	s	r	r	r	i^a^	s^a^	s^a^	s	s	NA, SXT, TE

52	chicken	*E. coli*	CTX-M group 1^+^	r	s	r	r	r	r	s^a^	s^a^	s	s	NA, SXT, TE

53	chicken	*E. coli*	CTX-M group 1^+^	r	s	r	r	r	i^a^	s^a^	s^a^	s	s	SXT, TE

58	chicken	*E. coli*	CTX-M-1	r	s	r	r	r	i^a^	s^a^	s^a^	s	s	GM, NA, SXT, TE

59	chicken	*E. coli*	CTX-M-1	r	s	r	r	r	i^a^	s^a^	s^a^	s	s	C, SXT, TE

60	chicken	*E. coli*	CTX-M-1	r	s	r	r	r	i^a^	s^a^	s^a^	s	s	SXT, TE

62	chicken	*E. coli*	CTX-M group 1^+^	r	s	r	r	r	i^a^	s^a^	s^a^	s	s	SXT, TE

63	chicken	*E. coli*	CTX-M group 1^+^	r	s	r	r	r	i^a^	s^a^	s^a^	s	s	SXT, TE

64	chicken	*E. coli*	CTX-M group 1^+^	r	s	r	r	r	i^a^	s^a^	s^a^	s	s	NA, SXT

65	chicken	*E. coli*	CTX-M group 1^+^	r	s	r	r	r	i^a^	s^a^	s^a^	s	s	TE

67	chicken	*E. coli*	CTX-M group 1^+^	r	s	r	r	r	i^a^	s^a^	s^a^	s	s	SXT, TE

68	chicken	*E. coli*	CTX-M group 1^+^	r	s	r	r	r	i^a^	s^a^	s^a^	s	s	SXT, TE

73	chicken	*E. coli*	CTX-M group 1^+^	r	s	r	r	r	i^a^	s^a^	s^a^	s	s	NA, TE

74	chicken	*E. coli*	CTX-M-1	r	s	r	r	r	i^a^	s^a^	s^a^	s	s	TE

76	chicken	*E. coli*	CTX-M group 1^+^	r	s	r	r	r	r	s^a^	s^a^	s	s	NA

78	chicken	*E. coli*	CTX-M group 1^+^	r	s	r	r	r	r	s^a^	s^a^	s	s	NA, TE

86	chicken	*E. coli*	CTX-M-1	r	s	r	r	r	i^a^	s^a^	s^a^	s	s	TE

87	chicken	*E. coli*	CTX-M-1	r	s	r	r	r	r	s^a^	s^a^	s	s	TE

88	chicken	*E. coli*	CTX-M-1	r	s	r	r	r	r	s^a^	s^a^	s	s	NA, SXT

91	chicken	*E. coli*	CTX-M group 1^+^	r	s	r	r	r	i^a^	s^a^	s^a^	s	s	NA, TE

92	chicken	*E. coli*	CTX-M-1	r	s	r	r	r	i^a^	s^a^	s^a^	s	s	S, TE

94	chicken	*E. coli*	CTX-M group 1^+^	r	s	r	r	r	r	s^a^	s^a^	s	s	TE

96	chicken	*E. coli*	CTX-M group 1^+^	r	s	r	r	r	i^a^	s^a^	s^a^	s	s	NA, SXT, TE

20	chicken	*E. coli*	CTX-M group 1/TEM^+^	r	s	r	r	r	i^a^	s^a^	s^a^	s	s	CIP, NA, SXT, TE

27	chicken	*E. coli*	CTX-M group 1/TEM^+^	r	s	r	r	r	i^a^	s^a^	s^a^	s	s	S, SXT, TE

32	chicken	*E. coli*	CTX-M-1 & TEM-1	r	s	r	r	r	r	s^a^	s^a^	s	s	SXT, TE

36	chicken	*E. coli*	CTX-M group 1/TEM^+^	r	i	r	r	r	i^a^	s^a^	s^a^	s	s	SXT, TE

82	chicken	*E. coli*	CTX-M group 1/TEM^+^	r	s	r	r	r	r	s^a^	s^a^	s	s	SXT, TE

84	chicken	*E. coli*	CTX-M-1 & TEM-1	r	s	r	r	r	r	s^a^	s^a^	s	s	NA, SXT, TE

85	chicken	*E. coli*	CTX-M group 1/TEM^+^	r	s	r	r	r	r	s^a^	s^a^	s	s	NA, SXT, TE

97	chicken	*E. coli*	CTX-M group 1/TEM^+^	r	s	r	r	r	r	s^a^	s^a^	s	s	SXT, TE

5	chicken	*E. coli*	SHV-12	r	s	r	i^a^	r	i^a^	i^a^	s^a^	s	s	-

2	chicken	*E. coli*	SHV-12	r	s	r	i^a^	r	i^a^	s^a^	s^a^	s	s	C, NA, TE

34	chicken	*E. coli*	SHV-12 & TEM-1	r	s	r	s^a^	r	s^a^	i^a^	s^a^	s	s	NA, SXT, TE

77	chicken	*E. coli*	SHV-12 & TEM-1	r	s	r	i^a^	r	i^a^	i^a^	s^a^	s	s	NA, SXT, TE

23	chicken	*E. coli*	TEM-52	r	s	r	i^a^	r	i^a^	s^a^	s^a^	s	s	NA

70	chicken	*E. coli*	TEM-52	r	s	r	r	r	i^a^	s^a^	s^a^	s	s	NA

1,006	mastitis milk	*E. coli*	CTX-M-14 & TEM-1	r	r	r	r	r	i^a^	s^a^	s^a^	s	s	C, GM, NA S, SXT, TE

^+^) not further characterized

s, sensitive; i, intermediate; r, resistant

^a^) It is known that many ESBL producers may appear susceptible or intermediate to oxyimino cephalosporins *in vitro *if CLSI criteria are applied strictly, but do not respond to the respective therapies. Consequently, for clinical reporting these results have to be corrected to "resistant".

AM, ampicillin; AMC, amoxicillin-clavulanic acid; CF, cephalothin; CXM, cefuroxime; CPD, cefpodoxime; CTX, cefotaxime; CAZ, ceftazidime; FEP, cefepime; FOX, cefoxitin; IPM, imipenem

C, chloramphenicol; CIP, ciprofloxacin; GM, gentamicin; NA, nalidixic acid; S, streptomycin; SXT, trimethoprim-sulfamethoxazole; TE, tetracycline

**Figure 1:**
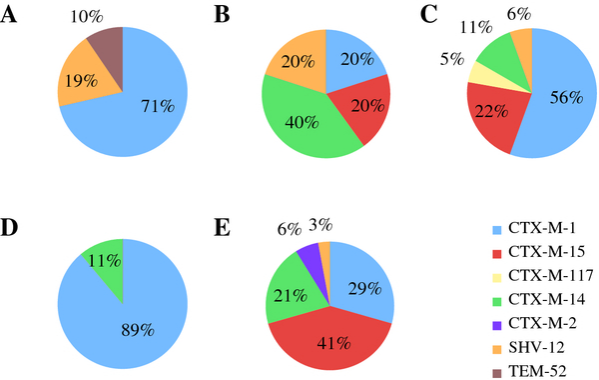
**Prevalence of different *bla*_ESBL _genes in *Enterobacteriaceae *isolated from food producing animals in Switzerland in comparison to isolates from healthy humans**. Prevalence of different *bla*_ESBL _genes in *Enterobacteriaceae *isolated from food producing animals in Switzerland in comparison to isolates from healthy humans. A, chickens; B, sheep; C, cattle; D, pigs; E, humans [17].

The ESBL-encoding genes of all isolates were further characterized by PCR. A total of 78 isolates (85.7%) produced CTX-M group 1 ESBLs while six isolates (6.6%) produced CTX-M group 9 enzymes. Five isolates (5.5%) were detected as producers of the SHV-ESBLs and 2 isolates (2.2%) exclusively produced TEM-type enzymes. Twenty-seven CTX-M carriers were additionally PCR-positive for *bla*_TEM _genes. Of the 91 ESBL producing isolates, 53 were selected for sequencing of the involved *bla *genes (Figure 1). Thirty-four isolates were CTX-M-1 producers, eight expressed additional TEM-1 and one isolate--from a pig-- additionally expressed a TEM-type enzyme, TEM-186 http://www.lahey.org/Studies/, never found before (nucleotide sequence accession number JN227084). Six isolates carried CTX-M-14 with TEM-1 and five isolates specified CTX-M-15, one of which producing additional TEM-1. One isolate from a calf produced TEM-1 in combination with CTX-M-117 http://www.lahey.org/Studies/, a novel CTX-M group 1 ESBL with an amino acid sequence never found before (nucleotide sequence accession number JN227085). Finally, two TEM-52 ESBL producers, and 5 SHV-12 carriers were found, three of the latter featuring additional TEM-1 (Table 2).

Besides the β-lactam resistances, the isolates were also tested for resistance to other classes of antibiotics. We found 76 (cattle: 13/17, pig: 6/9, sheep: 5/5, chicken: 51/62, milk: 1/1) out of 91 isolates resistant to tetracycline (83.5%), 59 isolates (cattle: 13/17, pig: 6/9, sheep: 2/5, chicken: 37/62, milk: 1/1) resistant to trimethoprim-sulfamethoxazole (64.8%), 43 isolates (cattle: 8/17, pig: 6/9, sheep: 4/5, chicken: 24/62, milk: 1/1) resistant to nalidixic acid (47.3%) and 31 (cattle: 13/17, pig: 6/9, sheep: 5/5, chicken: 6/62, milk: 1/1) resistant to at least one aminoglycoside (34.0%). Furthermore, 20 isolates (cattle: 11/17, pig: 2/9, sheep: 4/5, chicken: 2/62, milk: 1/1) showed resistance against chloramphenicol (22.0%), and 18 isolates (cattle: 7/17, pig: 3/9, sheep: 3/5, chicken: 4/62, milk: 1/1) showed resistance against ciprofloxacin (19.8%). One isolate from chicken faeces showed only resistance to ß-lactam-antibiotics, and none of the tested isolates was resistant to imipenem (Table 2).

## Discussion

Recently, an increase in studies, carried out in different countries, and describing the prevalence and characteristics of ESBL producing *Enterobacteriaceae *in cattle for example [2,15,18,19] and in pigs and chicken for example [20-25] were published. Moreover, some studies describing ESBL producing *Enterobacteriaceae *in salads [6], in meat [13,14] and in raw milk [12] are available. Since there seem to be geographical variations in the occurrence of different ESBL variants (e.g. CTX-M-9 in Spain as opposed to CTX-M group 1 in the Northern European countries [26]), it is therefore important to have a detailed overview based on geographical distribution and this knowledge was so far limited in Switzerland. Therefore, the present study provides further data concerning healthy animals (among them about sheep for the first time in literature), minced meat and bulk tank milk samples in Switzerland.

The high ESBL occurrence determined for all investigated animals in this study is surprising, given the fact that Switzerland is a country with a strict policy of antibiotic use [27]. Nevertheless, one reason could be the use of β-lactams--and even 4th generation cephalosporins--in veterinary medicine [28,29]. Another reason could be co-selection of multiple resistance mechanisms through the use of various antibiotics, due to the fact that resistance genes for aminoglycosides, tetracycline and trimethoprim-sulfametoxazole are frequently placed on single conjugative plasmids, as is often also the case with *bla*_ESBL _genes [4,26]. For food producing animals very limited data on the occurrence of ESBL producing *Enterobacteriaceae *had been available before in Switzerland, but there is a study about CTX-M producers in Swiss patients [30], and a recent report about occurrence of ESBL carriers in the healthy Swiss human population (5.8%) [17]. This is lower than the rates from food animals (8.6% to 63.4%) presented in this study. These frequencies primarily imply a reservoir of ESBL producers in farm animals. Supporting this view, a study from the Netherlands described the same CTX-M-type in chicken meat and humans [31]. In Switzerland, these findings cannot be confirmed because of the predominance of CTX-M-1 in animals and the predominance of CTX-M-15 in humans [17,30]. The predominance of CTX-M group 1 enzymes and the rare prevalence of CTX-M group 9, as seen in our study, has also recently been described in strains from healthy food-producing animals in Denmark, Portugal and France [8,32,33]. All of the TEM enzymes co-expressed alongside with CTX-M ESBLs were broad-spectrum β-lactamases--mostly TEM-1--conveying no ESBL phenotype. In contrast, two strains, expressing TEM enzymes exclusively, featured the TEM-ESBL TEM-52 (Table 2). In other countries TEM-ESBLs are much more frequently found in animals, especially in chickens [21,34].

Given the relatively high occurrence of ESBL producers in fecal samples from animals in our study, it is striking, that no ESBL producers could be found in either bulk tank milk or beef and pork minced meat. We hypothesise that the very high hygiene standards for slaughtering together with the selection of the raw meat for minced meat production and the quality based prizing system of bulk tank milk in Switzerland could be the reason for this favourable situation.

## Conclusion

The occurrence of ESBL producing *Enterobacteriaceae *in the fecal microflora of farm animals represents an obvious risk for contamination of raw food products from animal origin. However, since no ESBL producers were found in the examined food samples and our data concerning the ESBL type distribution in animals compared to healthy human carriers do not correlate well, animal food products can hardly be the major vector for ESLB carriage in the human population in Switzerland. Nevertheless, due to the generally high ESBL occurrence in food animals in Switzerland prudent use of antibiotics in veterinary medicine and strict hygiene measures during slaughtering and milking still remain important.

Finally, on the basis of the massive CTX-M-1 predominance in animals in our study compared to its relatively low frequency in healthy humans, further investigational efforts into the origin of the unexplained high occurrence of CTX-M-15 in the human population are warrented.

## Methods

### Sampling

Fecal samples were collected in October 2009 and from November 2010 to March 2011 from 334 healthy food-producing animals at slaughter in Switzerland: 59 pigs (57 fattening pigs, 2 piglets), 124 cattle (63 calves, 26 young cows, 18 fattening bulls, 10 cows and 7 bullocks) and 58 sheep (40 lambs, 18 sheep older than one year). To prevent sample clustering, at most two samples per farm were taken. The farms are distributed throughout Switzerland (16 cantons). Sampling was done with one swab per animal at a big EU-approved slaughterhouse (on average 1,000 pigs, 800 cattle, 60 sheep per day). Furthermore, 93 fecal samples of chicken were collected at the entry of a big EU-approved poultry slaughterhouse (on average 50,000 animals per day) from the crates of 93 poultry flocks (approximately 6,000 chicken per flock) distributed throughout Switzerland (14 cantons). Afterwards the swabs were put into an empty sterile tube, transported to the lab and processed within 6 hours of collection.

A total of 104 fresh minced meat samples (55 beef, 15 pork, 9 beef/pork, 3 veal, 3 beef/veal/pork, 2 lamb, 2 beef/lamb, and 15 of unknown origin) collected at 20 different days from a big meat processing plant (67.3% of the samples), which is supplying minced meat to retail stores and covers about 50% of the Swiss market and from local butcher shops (32.7% of the samples) were investigated.

Finally, 100 raw milk samples, representing bulk tank milk of 100 different dairy farms, were collected in April 2011 at a big dairy manufacturing plant in Switzerland. Furthermore, 67 *E*. *coli *isolates from cattle *E. coli *mastitis milk were investigated.

### Microbiological analysis

About 1 g of each fecal sample was enriched in 10 ml EE broth (BD, Franklin Lakes, USA) for 24 hours at 37°C. Moreover, 10 ml of the milk or 10 g of the meat samples were enriched for 24 hours at 37°C in 100 ml of EE Broth. Thereafter the enrichment was streaken onto Brilliance ESBL agar (Oxoid, Hampshire, UK), which was incubated at 37°C for 24 hours under aerobic conditions. All grown colonies of different color and/or morphology were selected and subcultured onto triple sugar iron (TSI) agar (BD, Franklin Lakes, USA) at 37°C for 24 hours. By the oxidase test, nonfermenters were discarded, and oxidase-negative colonies were subjected to identification by API ID 32 E (bioMérieux, Marcy 1'Etoile, France). Some isolates, yielding doubtful results, were subjected to genetic identification based on sequencing of 16S rRNA and *rpoB *gene fragments [35].

### Antimicrobial susceptibility testing and ESBL detection

All isolates were subjected to susceptibility testing against 17 antimicrobial agents by the disc diffusion method according to CLSI protocols and the results were evaluated according to CLSI criteria [36]. The antibiotics (Becton Dickinson, Sparks, MD USA) tested were: ampicillin (AM), amoxicillin/clavulanic acid (AMC), cephalothin (CF), cefuroxime (CXM), cefpodoxime (CPD), cefotaxime (CTX), ceftazidime (CAZ), cefepime (FEP), cefoxitin (FOX), imipenem (IMP), chloramphenicol (C), ciprofloxacin (CIP), gentamicin (GM), nalidixic acid (NA), streptomycin (S), trimethoprim-sulfamethoxazole (SXT), tetracycline (TE). The AMC disc was placed between those containing CPD and CAZ, and the resulting synergy effects were documented. The isolates, which showed a synergy effect between AMC and CPD and/or AMC and CAZ, were then confirmed as ESBL producers on Muller-Hinton agar plates using E-Test-ESBL strips containing cefotaxime, cefepime or ceftazidime each alone and in combination with clavulanic acid (bioMérieux, Marcy 1'Etoile, France) according to the manufacturer's recommendations.

### Characterization of β-lactamases

Bacterial isolates confirmed for producing ESBLs were further analysed by PCR. DNA was extracted by a standard heat lysis protocol. Thereafter, five specific primer sets (custom-synthesized by Microsynth, Balgach, Switzerland) were used to search for *bla*_TEM_, *bla*_SHV _and *bla*_CTX-M _genes [37-39].

### PCR amplification and sequencing of *bla *open reading frames (ORF)

The ESBL-encoding genes of the isolated ESBL producers from cattle (17), sheep (5), pigs (9) and mastitis milk, as well as of 21 of the 59 isolates from chickens were sequenced. To be able to sequence the whole ORFs, five PCR/sequencing primers were used. Two per *bla *family were the respective screening primers (see above), three per *bla *family were designed newly (this study), and custom-synthesized by Microsynth (Balgach, Switzerland). Primers were: primer 1_1 _forward 5'-AAACACACGTGGAATTTAGGG-3' primer 2_1 _forward, 5'-AAAAATCACTGCGCCAGTTC-3' [39], primer 3_1 _reverse, 5'-AGCTTATTCATCGCCACGTT-3' [39], primer 4_1 _reverse, 5'-CCGTCGGTGACGATTTTAGCC-3', primer 5_1 _reverse, 5'-CCGATGACTATGCGCACTGGG-3', for *bla*_CTX-M-group1_; 1_2 _forward, 5'-TTTTGCCGTACCTGCGTACCC-3', primer 2_2 _forward, 5'-CGACGCTACCCCTGCTATT-3' [39], primer 3_2 _reverse, 5-CCAGCGTCAGATTTTTCAGG-3' [39], 4_2 _reverse, 5'-CCGTGGGTTACGATTTTCGCC-3', 5_2 _reverse, 5'-TTGGTCCAGAAAAAAGAGCGG-3' for *bla*_CTX-M-group2 _and 1_9 _forward, 5'-TGATGTAACACGGATTGACCG-3' 2_9 _forward, 5'-CAAAGAGAGTGCAACGGATG-3' [39], 3_9 _reverse, 5'-ATTGGAAAGCGTTCATCACC-3' [39], 4_9 _reverse, 5-AAACCAGTTACAGCCCTTCGG-3' and 5_9 _reverse, 5-TGGAGCCACGGTTGATGAGGG-3' for *bla*_CTX-M-group9_. Primer pairs for PCR were: 1-3, 1- 4, 1-5, 2-4 and 2-5 and PCR-conditions comprised initial denaturation at 94°C for 15 sec, followed by 35 cycles each including steps for denaturation at 94°C for 30 sec, annealing at 53°C for 30 sec and elongation at 72°C for 30 sec, followed by a final extension at 72°C for 7 min. For sequencing of TEM genes the same primers and PCR conditions as before were used [38], whereas for the SHV genes sequencing primers were used as described previously [40]. PCR-conditions for *bla*_SHV _genes were the same as those for TEM genes [38]. Resulting amplicons were purified using the PCR Purification Kit (QIAGEN, Courtaboeuf, France) according to the manufacturer's recommendations. Custom-sequencing was performed at Microsynth (Balgach, Switzerland) and the nucleotide and protein sequences were analyzed with Codon Code Aligner V. 3.7.1.1. For database searches NCBI at the BLASTN program package http://www.ncbi.nlm.nih.gov/blast/ was used.

### Statistical analysis

95 percent confidence intervals were calculated using R software http://www.R-project.org/.

## Authors' contributions

NG and HH were responsible for isolation and characterization of strains and drafted the manuscript. RS and HH designed the study and edited the manuscript. All authors read, commented on, and approved the final manuscript.
